# The JNK Signaling Pathway Regulates Seizures Through ENT1 in Pilocarpine‐Induced Epilepsy Rat Model

**DOI:** 10.1111/cns.70190

**Published:** 2024-12-25

**Authors:** Shun Liu, Zhong Luo, Fangjing Li, Lijia Zhang, Mingxiang Xie, Juan Yang, Zucai Xu

**Affiliations:** ^1^ Department of Neurosurgery Affiliated Hospital of Zunyi Medical University Zunyi Guizhou China; ^2^ The Collaborative Innovation Center of Tissue Damage Repair and Regeneration Medicine of Zunyi Medical University Zunyi Guizhou China; ^3^ Department of Neurology Affiliated Hospital of Zunyi Medical University Zunyi Guizhou China

**Keywords:** adenosine, C‐Jun N‐terminal kinase, epilepsy, equilibrium nucleoside transporter 1, glutamate

## Abstract

**Objective:**

The study investigates whether the expression and function of ENT1 can be regulated by inhibiting the JNK signaling pathway, thereby altering the levels of extracellular adenosine and glutamate in neurons, and subsequently affecting the progression of epilepsy.

**Methods:**

The adult male SD rats were randomly divided into four groups: EP + SP600125 group, EP + DMSO group, EP group, and normal control group. The expression levels of ENT1, p‐JNK, and JNK in the hippocampus of rats from each experimental group were detected using Western blotting technology. The expression and localization of ENT1 and p‐JNK in the CA1, CA3, and DG areas of the hippocampus were detected by immunohistochemical staining and immunofluorescence staining. Microdialysis combined with liquid chromatography‐mass spectrometry was used to determine the concentrations of adenosine and glutamate in the extracellular fluid of hippocampus in each experimental group.

**Results:**

This study showed that the JNK‐specific inhibitor SP600125 could reduce ENT1 expression and seizure intensity in experimental rats. Statistical analysis confirmed that adenosine and glutamate levels in the extracellular fluid of the hippocampus increased significantly after seizures in rats, and the JNK‐specific inhibitor SP600125 could increase adenosine levels in the extracellular fluid but decrease glutamate levels.

**Significance:**

The JNK‐specific inhibitor SP600125 can specifically inhibit the JNK signaling pathway and reduce the expression of ENT1 transporter. The mechanism is related to the transport of adenosine from the extracellular space to the intracellular space by ENT1 during epileptic states. Inhibition of ENT1 can increase the concentration of adenosine in the extracellular fluid of the hippocampus. The increase in adenosine concentration stopped glutamate from being released and reduced the amount of glutamate in the outside of the cell.

## Graphical abstract

The JNK‐specific inhibitor SP600125 can specifically inhibit the JNK signaling pathway, reduce the expression of the ENT1 transporter, and reduce the seizure intensity in experimental rats. The mechanism is related to the transport of adenosine from the extracellular space to the intracellular space by ENT1 during epileptic states. Inhibition of ENT1 can increase the concentration of adenosine in the extracellular fluid of the hippocampus. The increase in adenosine can inhibit the release of glutamate by acting on the A1 receptor of the presynaptic membrane.

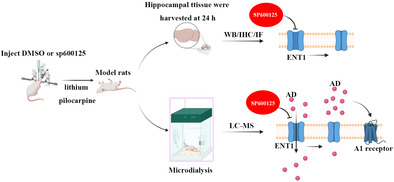

## 1 Introduction

Epilepsy is a common neurological disorder affecting around 50 million people worldwide [1, 2]. Among these, epilepsy cannot be effectively controlled in many patients even after drug treatment [3, 4]. Due to its recurrent attacks and difficult cure, it often causes serious damage to the life and work of patients and has become a prominent and thorny public health problem worldwide [5, 6]. Therefore, clarifying its formation mechanism and designing specific antiepileptic drugs on this basis has become the focus and hotspot of neuroscience.

Adenosine is an important neurotransmitter. It mediates the activities of corresponding receptors and acts on mechanisms such as neuroimmune response, blood–brain barrier permeability, synaptic transmission, neuronal excitability, and cellular bioenergy [7, 8]. Regulation of adenosine levels can inhibit seizures and have anticonvulsant effects [9, 10, 11].

Anderson CM and others confirmed through experiments that the equilibrium nucleoside transporter 1 (ENT1) could balance the concentration of adenosine inside and outside cells and have a certain impact on the expression and function of the adenosine A1 receptor [12, 13, 14, 15]. ENT1 is widely expressed in human brain tissue in regions such as the amygdala, caudate nucleus, hippocampus, and thalamus [16, 17]. Currently, there is no detailed study of the transport dynamics and specific mechanism of ENT1 interaction with adenosine in the epileptic state.

C‐Jun N‐terminal kinase (JNK) is one of the three main MAPK signaling pathways in animals [18, 19]. It has been implicated in the study of Alzheimer's disease, type 2 diabetes, and retinal degeneration [20, 21, 22]. The relationship between this pathway and epilepsy was confirmed as early as 2006: after epilepsy, the JNK signaling pathway was significantly activated in neurons [23]. In addition, during seizures, inhibiting activation of the JNK pathway can reduce glutamate release by reducing the generation of epileptiform discharges [24]. Further studies have shown that activation of the JNK signaling pathway plays a regulatory role in the expression and function of ENT1 in murine myeloid leukemia cells and other models [25, 26].

In this experiment, we established a lithium pilocarpine model of acute epilepsy in rats. The intervention model was performed by stereotactic injection of DMSO and the JNK‐specific inhibitor SP600125. We explored whether the expression and function of ENT1 could be regulated by inhibiting the JNK signaling pathway to change the levels of adenosine and glutamate outside the neurons and then affect the occurrence and development of epilepsy. This study may help us elaborate on the role of “JNK signaling pathway regulating ENT1” in the seizure model.

## 2 Materials and Methods

### 2.1 Animal Models and Drug Interventions

Adult male Sprague–Dawley (SD) rats weighing 300–350 g were selected as experimental subjects for the experiment. These rats were obtained from the breeding colony of Hunan Silaike Jingda Experimental Animal Co. Ltd. Each cage housed four rats and was maintained at a constant standard culture environment (ventilated room, 25°C ± 1°C, 60% ± 5% humidity, 12 h light/dark cycle), with free access to standard water and food (SCXK2019‐0004). All procedures followed the “Guiding Principles in the Care and Use of Animals” (China).

Adult male SD rats were randomly divided into four groups: the EP + SP600125 group (SP600125 was dissolved in 100% DMSO to 0.3 mg/μL, and injected 5 μL into the bilateral hippocampus by stereotactic instrument, *n* = 6), EP + DMSO group (5 μL of 100% DMSO was injected into the bilateral hippocampus by stereotactic instrument, *n* = 6), EP group (5 μL of artificial cerebrospinal fluid was injected into the bilateral hippocampus by rat brain stereotactic instrument, *n* = 6), and normal control group (*n* = 6) [27].

One hour after stereotactic injection of DMSO, SP600125, or ACSF (artificial cerebrospinal fluid, 147 mM NaCl, 2.8 mM KCl, 1.2 mM CaCl2, 0.8–1.2 mM MgCl2, pH = 7.4 adjusted with NaOH) into rats, we established a lithium pilocarpine model of acute epilepsy in rats. Lithium chloride (127 mg/kg) was injected into the rats, followed 18–24 h later by injection of atropine sulfate (1 mg/kg). Thirty minutes later, pilocarpine (50 mg/kg) was administered intraperitoneally. The rats exhibited seizure activity classified as stage IV or V on the Racine scale [28]. Status epilepticus (SE) lasted for 45 min and was terminated by intraperitoneal injection (i.p.) of atropine sulfate (1 mg/kg) and diazepam (10 mg/kg). The normal control group received the same doses of lithium chloride and atropine sulfate, but pilocarpine was replaced by 0.9% saline. Tissues were collected 24 h after successful modeling.

### 2.2 Stereotactic Injection

First, the rats were anesthetized with pentobarbital (50 mg/kg, i.p.) and then DMSO, SP600125, or ACSF was stereotactically injected into the bilateral hippocampus of the experimental rats (−3.72 mm AP and ± 2.00 mm ML, 2.80 mm DV) [29]. Each hippocampus was injected with the solution at a rate of 1 μL/ min for 5 min. After sealing the injection with bone wax, the injection area was disinfected and sutured layer by layer. Prevent infection by subcutaneous injection of 800,000 U penicillin.

### 2.3 Western Blot

Rats were anesthetized with pentobarbital (50 mg/kg, i.p.) and the brain was quickly removed. The hippocampus and cortex of the experimental group rats were then rapidly dissected and frozen. Total protein concentration was measured using the enhanced BCA protein detection kit (Beyotime, Haimen, China) according to the instructions. Equal amounts of protein were separated by 10% SDS‐PAGE, and transferred to polyvinylidene difluoride (PVDF) membranes. The PVDF membrane was sealed with 5% skim milk for 1 h at room temperature and incubated overnight at 4°C with the appropriate primary antibodies, including Anti‐phospho‐JNK antibody (rabbit monoclonal antibody, 1:2000, Abcam, ab124956), Anti‐JNK antibody (rabbit monoclonal antibody, 1:2000, Zenbio 344,554), Anti‐ENT1 antibody (rabbit monoclonal antibody, 1:200, Abcam, ab135756), GAPDH antibody (mouse monoclonal antibody, 1:2000, Proteintech, 1E6D9). The GAPDH antibody is used as an internal reference protein. After the first incubation, the membranes were removed and incubated again with the second antibody at 25°C for 1 h, including HRP Anti‐rabbit IgG (1:5000, Zenbio 511,203), HRP Anti‐mouse IgG (1:5000, Zenbio 701,051). The protein bands were then visualized. Super Signal West Pico Chemiluminescent HRP substrate (Rockford, IL, USA) was used for this step. Finally, the data were analyzed using Quantity One software (Bio‐Rad Laboratories, Hercules, CA, USA) [30].

### 2.4 Immunohistochemistry Staining

Randomly selected paraffin sections were incubated with an anti‐phospho‐JNK antibody (rabbit monoclonal antibody, 1:200, Abcam, ab124956) or Anti‐ENT1 antibody (rabbit monoclonal antibody, 1:200, Abcam, ab135756) overnight at 4°C. The following day, staining was performed using the DAB chromogenic kit, and the staining was terminated when light brown particles were observed under the microscope and stored properly for further observation after drying.

### 2.5 Immunofluorescence Staining

Randomly selected frozen sections were incubated overnight at 4°C with a mixture of mouse anti‐GFAP (1:50; Santa Cruz Biotechnology, USA, sc‐71,143), guinea pig anti‐microtubule‐associated protein 2 (MAP2) (1:50, Sysy, Goettingen, Germany, 188,004), anti‐phospho‐JNK antibody (1:50) or Anti‐ENT1 antibody (1:50). After overnight incubation, the sections were mixed with Alexa Fluor 488‐conjugated goat anti‐rabbit IgG (1:100, Jackson ImmunoResearch, USA), Alexa Fluor 594‐conjugated goat anti‐mouse IgG (1:100, Jackson ImmunoResearch, USA), and Alexa Fluor 405‐conjugated goat anti‐guinea pig IgG (1:100, Jackson ImmunoResearch, USA). The sections were then counterstained with DAPI (4′,6‐diamidino‐2‐phenylindole, 1:10,000 dilution, Sigma‐Aldrich, D9542) for 20 min, sealed in glycerol, and prepared as observation slides. Finally, they were observed under a laser scanning confocal microscope at 40× magnification [31].

### 2.6 Microdialysis Combined With Liquid Chromatography‐Mass Spectrometry (LC‐MS)

Rats were anesthetized with pentobarbital (50 mg/kg, i.p.) and the guiding catheter (CXG‐4, Eicom, Japan) was stereotactically implanted into the hippocampus of the experimental rats (−3.72 mm AP and ± 2.00 mm ML, 2.80 mm DV). The guiding catheter was then fixed with two screws and dental adhesive, and sealed with a matching catheter core (CXD‐4, Eicom, Japan). A subcutaneous injection of 800,000 U penicillin was given to prevent infection. After 24 h of successful catheterization, normal rat cerebrospinal fluid was collected, and then DMSO, SP600125, or ACSF was stereotactically injected into the bilateral hippocampus of the experimental rat. One hour later, a lithium pilocarpine model of acute epilepsy in rats was established. The following steps were performed 24 h after successful modeling.

**FIGURE 1:**
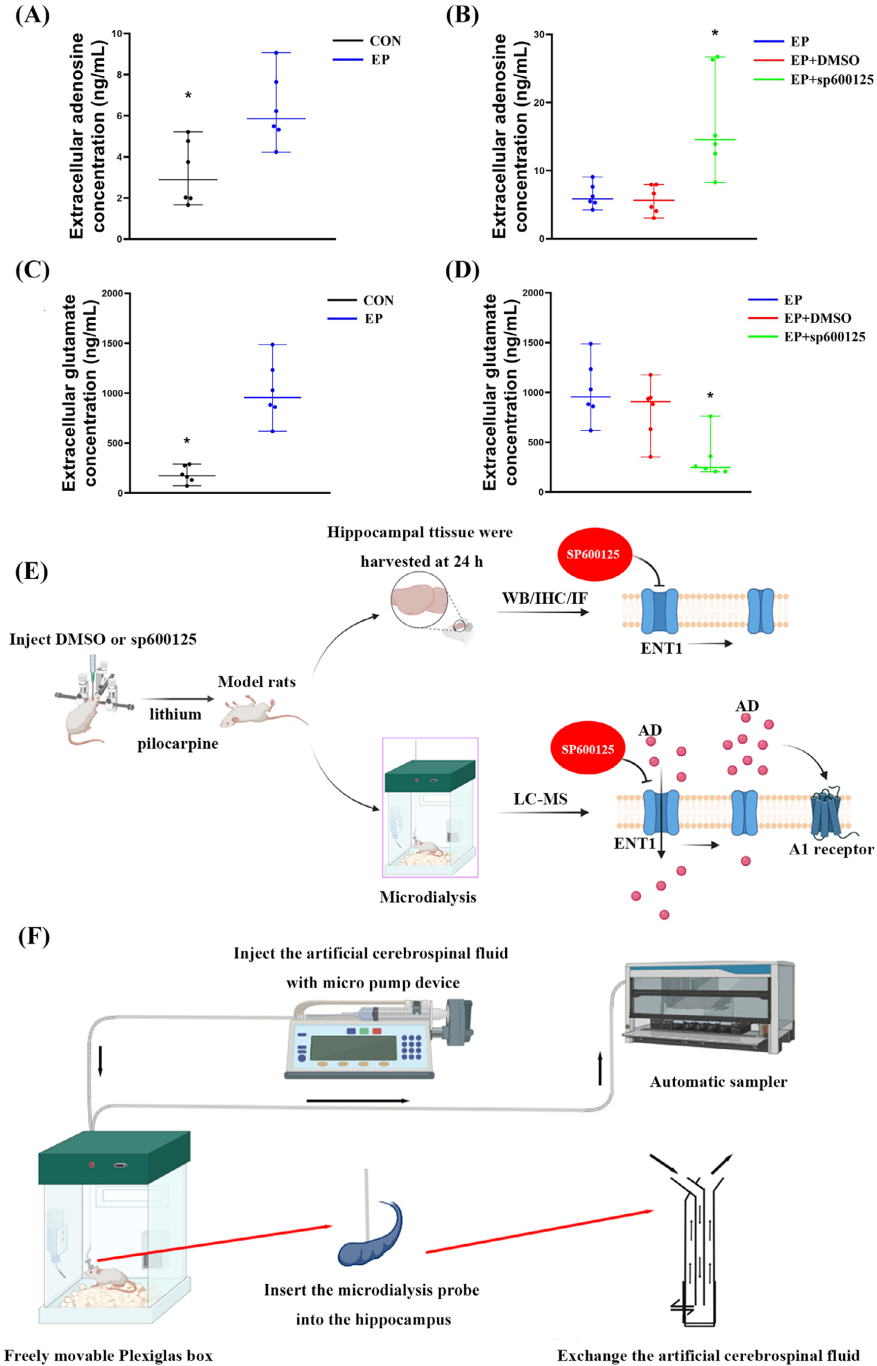
Analysis of extracellular adenosine (A) or glutamate (C) concentration in the hippocampus of six rats before and after epileptic seizures, and differences in adenosine (B) or glutamate (D) levels between each group. Adenosine and glutamate levels in the extracellular fluid of the hippocampus increased significantly after seizures in rats, and the JNK‐specific inhibitor SP600125 can specifically inhibit the JNK signaling pathway to increase adenosine levels in the extracellular fluid. The increase in adenosine can inhibit the release of glutamate by acting on the A1 receptor of the presynaptic membrane (E). The experimental rat was placed in a freely movable Plexiglas box, connected to the micropump device, and continuously perfused with ACSF at the flow rate and collected using an automatic sampler (F). *Compared with the EP group, *p* < 0.05.

The experimental rat was placed in a freely movable Plexiglas box (Eicom, Japan). After adapting to the environment, the rat was lightly anesthetized with 2% isoflurane oxygen, and then the microdialysis probe (CX‐I‐ 4‐2, Eicom, Japan) was inserted into the pre‐embedded catheter. The micropump device (ESP‐32, Eicom, Japan) was connected and ACSF was continuously perfused at a flow rate of 2 μL/min. After 2 h, four consecutive 60 μL samples were collected (Figure 1F).

The automated sampler (ESA model 542) was used to collect a predetermined 60 μL of dialysate, which was analyzed by high‐performance liquid chromatography with an electrochemical detector (ESA—Coulochem III). Detection of glutamate: The chromatographic column was Thermo Hypersil GOLD HILIC 100 × 2.1 mm, 1.9 μm. The mobile phase consisted of the following (mM): aqueous formic acid solution (0.1%), formic acid solution, and acetonitrile (0.1%). The flow rate was 0.45 mL/min. Detection of adenosine: The chromatographic column was Welch XB C18 150 × 4.6 mm, 5 μm. The mobile phase included the following (mM): ammonium formate and pure methanol. The flow rate was 1.0 mL/min. The recovery rate of the microdialysis probe could be calculated by dividing the concentration after dialysis by the known concentration before dialysis.

The conditions of mass spectrometry: positive ion scanning mode; electrospray voltage: 4000 V; ion source temperature: 350°C; collision gas: high‐purity argon; sheath gas and auxiliary gas: high‐purity nitrogen. Accurately weigh 50 μL of samples, add 50 μL of 0.1% formic acid water, vortex mix, and sample injection analysis.

### 2.7 Statistical Analysis

The Shapiro–Wilk test was used to inspect the normality of all the data. Data with a normal distribution were reported as mean ± standard deviation. Once the data had been found to satisfy the assumptions of normality and homogeneity of variance, the Student's *t*‐test and one‐way analysis of variance were employed. When the data exhibited a normal distribution but did not conform to the homogeneity of variance, the Kruskal–Wallis test was used. Data that did not follow a normal distribution were expressed as the median with a 95% confidence interval and evaluated using a rank sum test. *p* < 0.05 (indicated by * in the figures) was considered significant. SPSS 18.0 software was used for analysis.

## 3 Results

### 3.1 Inhibiting Activation of the JNK Signaling Pathway Can Reduce Seizures in Rat Model

**FIGURE 2:**
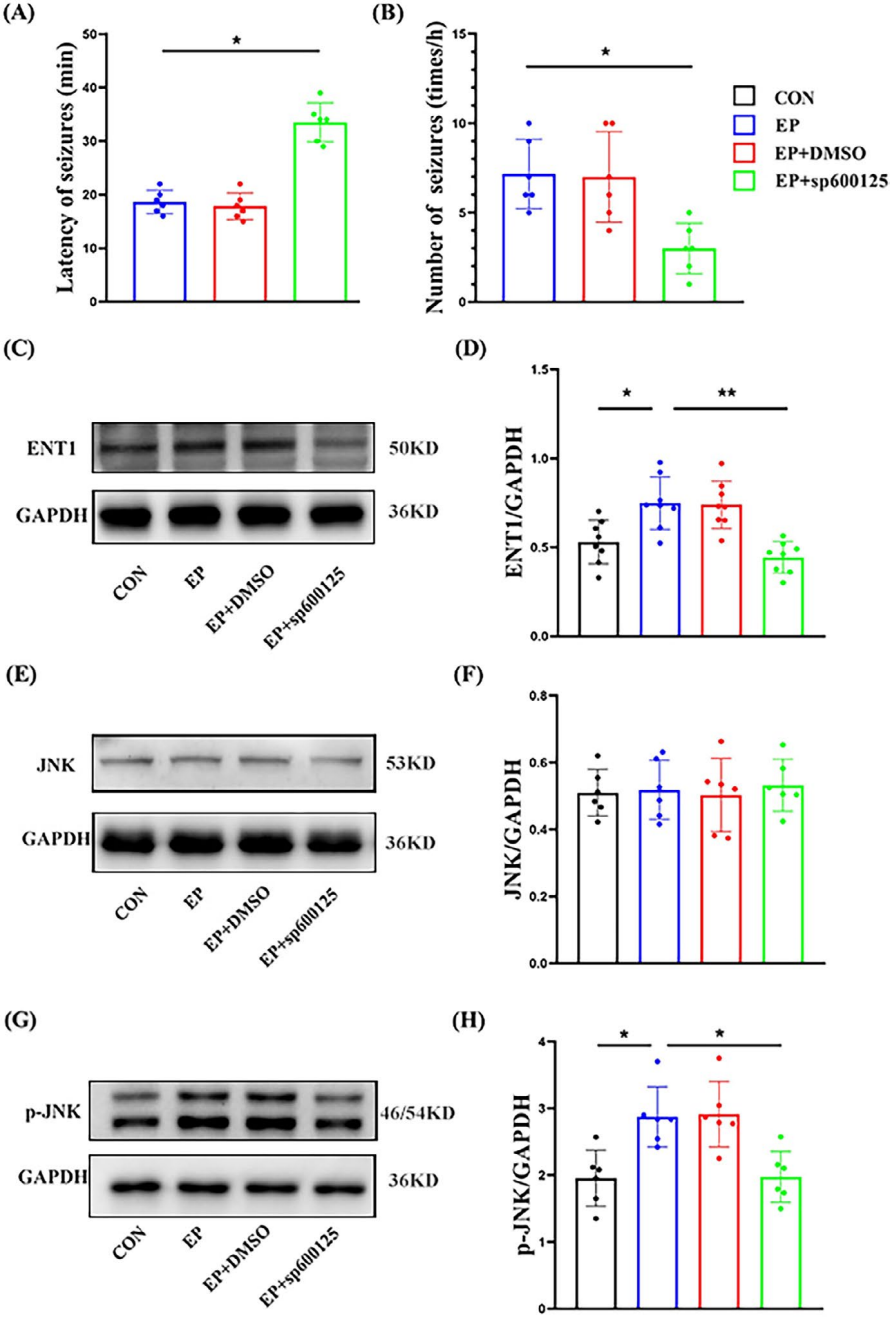
The incubation period of the first epileptic seizure (A) and the seizure frequency within 1 hour in rats (B). The typical band of the ENT1 or GAPDH (C), the JNK or GAPDH (E), and the p‐JNK or GAPDH (G). The amount of ENT1 protein (D), JNK protein (F), and p‐JNK protein (H) was quantified by densitometry and corrected for the amount of GAPDH in the corresponding lysate. *Compared with the EP group, *p* < 0.05; **compared with the EP group, *p* < 0.01.

The incubation period of the first epileptic seizure was significantly prolonged in the SP600125 group, and the number of seizures within 1 h was significantly lower than that in the EP group (*p* < 0.05). No significant difference between the DMSO and EP groups (*p* > 0.05) (Figure 2A,B).

### 3.2 Inhibition of the JNK Signaling Pathway Can Reduce the Expression of ENT1 and p‐JNK

Western blot was used to detect ENT1 protein expression in the hippocampus of each group. The expression levels of ENT1 protein increased significantly in the EP and DMSO groups, while the expression levels of ENT1 protein in the hippocampus of the SP600125 group were significantly lower than that of the EP group (*p* < 0.01). No significant difference existed in the levels of ENT1 protein expressed between the DMSO and EP groups (*p* > 0.05) (Figure 2C,D).

Western blot was used to detect JNK protein expression in the hippocampus of each group. Because SP600125 had no significant effect on the expression levels of total JNK protein, there was no significant difference existed between each group (*p* > 0.05) (Figure 2E,F).

Western blot was used to detect p‐JNK protein expression in the hippocampus of each group. The expression levels of p‐JNK protein increased significantly in the EP and DMSO groups, while the expression levels of p‐JNK protein in the hippocampus of the SP600125 group were significantly lower than that of the EP group (*p* < 0.05). No significant difference existed in the levels of p‐JNK protein expressed between the DMSO and EP groups (*p* > 0.05) (Figure 2G,H).

### 3.3 Inhibition of the JNK Signaling Pathway Can Reduce the Number of ENT1‐Positive Cells and p‐JNK‐Positive Cells in the Hippocampus

**FIGURE 3:**
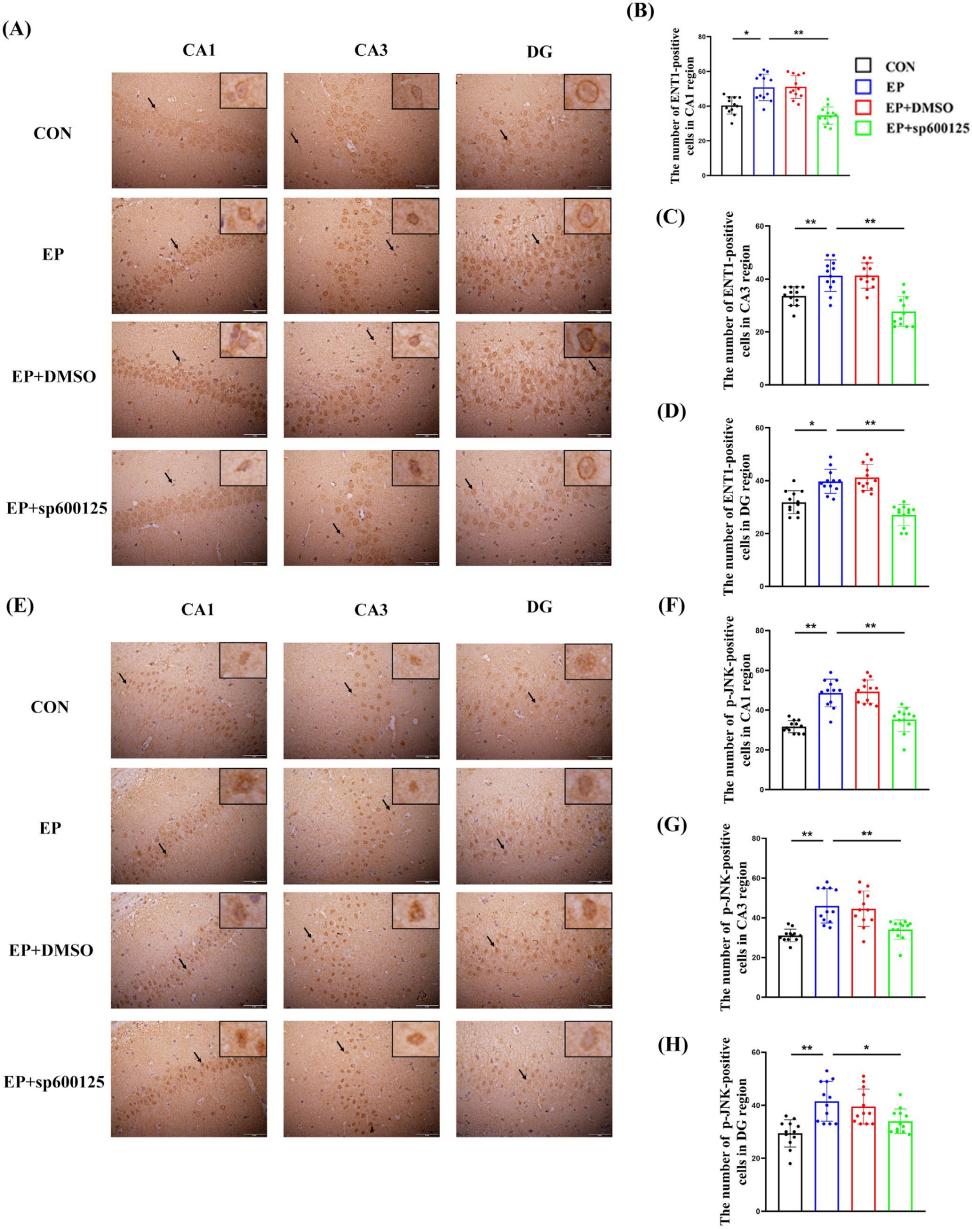
Expression of ENT1 protein (A) or p‐JNK protein (E) in the hippocampus (CA1, CA3, and DG areas) in each group (200×). Analysis of the number of ENT1‐positive cells in the CA1 (B), CA3 (C), and DG areas (D) and p‐JNK‐positive cells in the CA1 (F), CA3 (G), and DG areas (H). *Compared with the EP group, *p* < 0.05; **compared with the EP group, *p* < 0.01.

Immunohistochemical staining was used to detect the expression of ENT1 protein in the hippocampus (CA1, CA3, and DG areas) in each group: ENT1 protein was mainly expressed on the cell membrane. The number of ENT1‐positive cells increased significantly in the EP and DMSO groups, while the number of ENT1‐positive cells in the hippocampus of the SP600125 group was significantly lower than that of the EP group (*p* < 0.05). There was no significant difference existed in the number of ENT1‐positive cells between the EP and DMSO groups (*p* > 0.05) (Figure 3A–D).

Immunohistochemical staining was used to detect the expression of p‐JNK protein in the hippocampus (CA1, CA3, and DG areas) in each group: p‐JNK protein was mainly expressed in the nucleus and part of the cytoplasm. The number of p‐JNK‐positive cells increased significantly in the EP and DMSO groups, while the number of p‐JNK‐positive cells in the hippocampus of the SP600125 group was significantly lower than that of the EP group (*p* < 0.05). There was no significant difference existed in the number of p‐JNK‐positive cells between the EP and DMSO groups (*p* > 0.05) (Figure 3E–H).

### 3.4 Inhibition of the JNK Signaling Pathway Can Reduce the Average Fluorescence Intensity of ENT1 and p‐JNK in the Hippocampus

**FIGURE 4:**
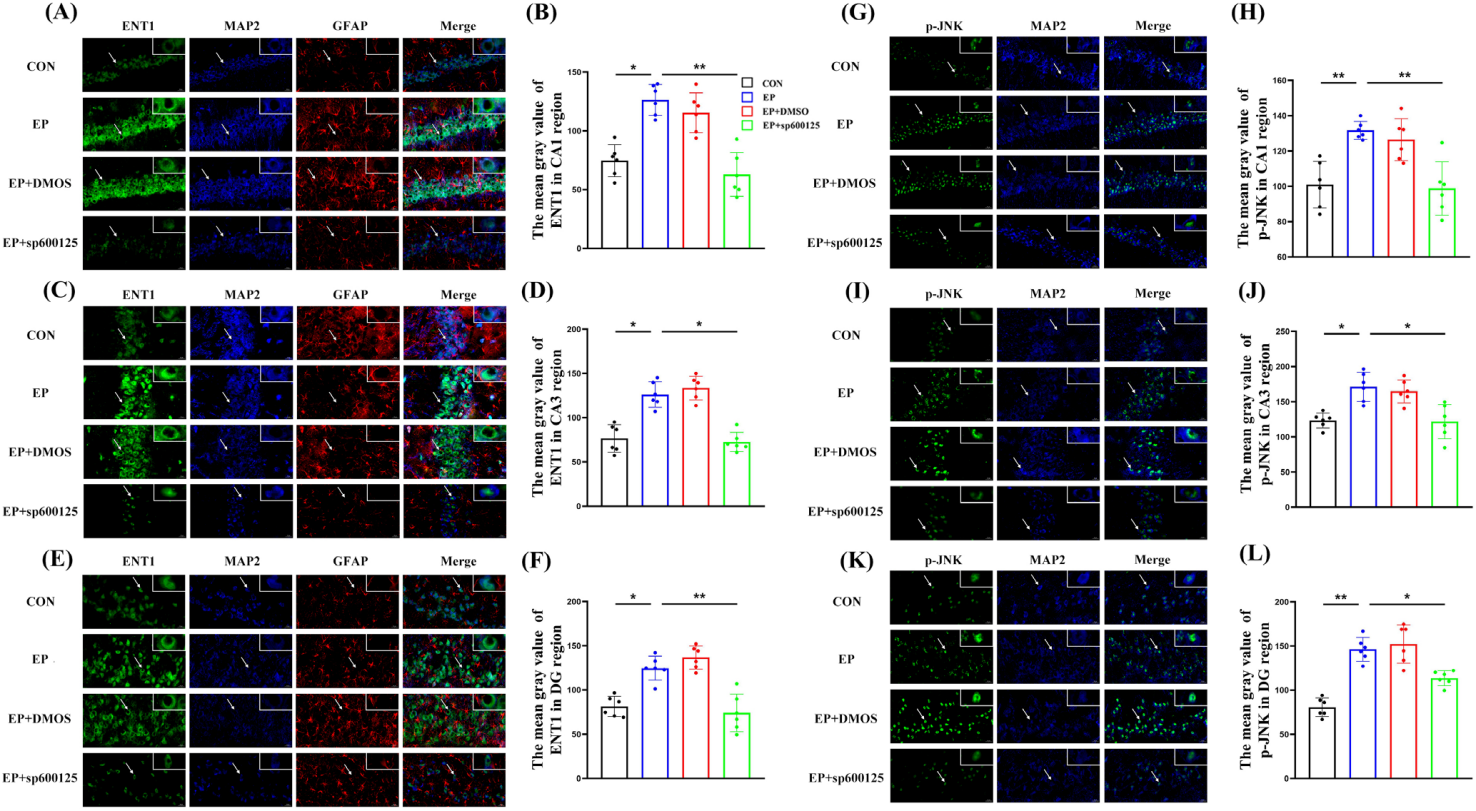
Expression levels of immunofluorescence detection and the statistics of the mean gray value of ENT1 in the CA1 (A, B), CA3 (C, D), and DG areas (E, F) (400×). Expression levels of immunofluorescence detection and the statistics of the mean gray value of p‐JNK in the CA1 (G, H), CA3 (I, J), and DG areas (K, L) (400×). *Compared with the EP group, *p <* 0.05; **compared with the EP group, *p* < 0.01.

Immunofluorescence staining was used to assess subcellular localization. ENT1 was coexpressed with the neuron dendritic‐specific marker MAP2 in the CA1, CA3, and DG areas of the hippocampus. The average fluorescence intensity of ENT1 increased significantly in the EP and DMSO groups, while it was lower in the SP600125 group, which was statistically significant compared to the EP group (*p* < 0.05). There was no significant difference existed in the average fluorescence intensity of ENT1 between the EP and DMSO groups (*p* > 0.05) (Figure 4A–F).

Immunofluorescence staining was used to assess subcellular localization. p‐JNK was coexpressed with the neuron dendritic‐specific marker MAP2 in the CA1, CA3, and DG areas of the hippocampus. The average fluorescence intensity of p‐JNK increased significantly in the EP and DMSO groups, while it was lower in the SP600125 group, which was statistically significant compared to the EP group (*p* < 0.05). There was no significant difference existed in the average fluorescence intensity of p‐JNK between the EP and DMSO groups (*p* > 0.05) (Figure 4G–L).

### 3.5 Inhibition of the JNK Signaling Pathway Can Increase the Concentration of Adenosine and Decrease the Concentration of Glutamate in the Extracellular Fluid of the Hippocampus

Extracellular fluid was collected from the rat hippocampus of conscious rats by microdialysis, and then the concentration of extracellular adenosine was determined by LC‐MS. By collecting the extracellular fluid from the hippocampus of six rats before and after the seizure, the results showed that the concentration of extracellular adenosine increased to varying degrees after seizure. The extracellular adenosine concentration increased in the EP and DMSO groups, but was significantly higher in the SP600125 group than in the EP group (*p* < 0.05). There was no significant difference existed between the EP and DMSO groups (*p* > 0.05) (Figure 1A,B).

Extracellular fluid was collected from the rat hippocampus of conscious rats by microdialysis, and then the concentration of extracellular glutamate was determined by LC‐MS. By collecting the extracellular fluid from the hippocampus of six rats before and after the seizure, the results showed that the concentration of extracellular glutamate increased to varying degrees after seizure. The extracellular glutamate concentration increased in the EP and DMSO groups, but was significantly lower in the SP600125 group than in the EP group (*p* < 0.05). There was no significant difference existed between the EP and DMSO groups (*p* > 0.05) (Figure 1C,D).

## 4 Discussion

As an important neurotransmitter, adenosine is mainly regulated by the nucleoside regulatory system. Adenosine acts on corresponding receptors and is involved in physiological activities such as the neuroimmune response, blood–brain barrier permeability, synaptic transmission, neuronal excitability, and cellular metabolism [7, 8]. The occurrence and development of epilepsy are closely related to the function of neurotransmitters. Under normal physiological conditions, excitatory and inhibitory neurotransmitters are in equilibrium and maintain the stability of neuronal cell membranes. When this balance is disturbed, disorders of the central nervous system occur, leading to diseases such as epilepsy, depression, and dementia [32, 33, 34]. Purine P1 receptors are mainly activated by adenosine and include four subtypes: A1, A2A, A2B, and A3 [7, 35]. Extracellular adenosine can inhibit the expression of presynaptic excitatory neurotransmitters and the hyperpolarization of the postsynaptic membrane by activating the A1 receptor, thus achieving antiepileptic effects [36]. The antiepileptic activity of adenosine is related to the increase in its extracellular concentration. Experiments have confirmed that the increase in extracellular adenosine can effectively inhibit the epilepsy kindling model and epilepsy induced by kainic acid and bicuculline [9], and the application of adenosine kinase (ADK) inhibitors and adenosine uptake inhibitors have some effect on the treatment of epilepsy [37]. As an inhibitory neurotransmitter, adenosine can also inhibit glutamate release by acting on the presynaptic membrane A1 receptor [38]. In the epilepsy model, inhibiting the activation of the JNK signaling pathway can reduce the release of glutamate to reduce the generation of epileptic‐like electrical activity [24].

The equilibration nucleoside transporter (ENT), encoded by the SLC29 gene, is a bidirectional passive nucleoside transporter located mainly at the cell membrane that can transport substrates along their concentration gradients [39, 40, 41]. Experiments have shown that ENT1 can not only balance the concentration of adenosine inside and outside cells but is also related to the function of the adenosine A1 receptor [13, 14]. Under normal physiological conditions, inhibition of ENT1 can lead to an increase in the level of extracellular adenosine surrounding brain neurons, while enhancement of ENT1 activity can increase the absorption of adenosine by neurons and decrease the level of extracellular adenosine surrounding neurons; that is, under normal physiological conditions, the transport trend of adenosine by ENT1 is from extracellular to intracellular [42]. When brain tissue is ischemic and hypoxic, ATP is largely converted to adenosine due to increased energy consumption. At this time, ENT1 mainly transports adenosine out of the cell, significantly increasing the extracellular adenosine levels [43]. However, by simulating the conditions of cellular ischemia and hypoxia in vitro, it has been confirmed that ENT1 still transferred adenosine from the extracellular space to the intracellular space [44]. Under pathological conditions, the trend of adenosine transport by ENT1 is uncertain. There is no detailed study of the trend of adenosine transport by ENT1 and its specific mechanism during the epileptic state. However, it is undeniable that ENT1 can affect the level of extracellular adenosine through various pathways and plays a key role in the occurrence and development of epilepsy [45, 46]. Therefore, the focus of this experiment is to elucidate the specific transport mechanism of adenosine through ENT1 after epilepsy.

In recent years, the JNK‐mediated phosphorylation signaling pathway has been identified as a new antiepileptic target [47, 48]. Deletion of its subtype has a certain neuroprotective effect in temporal lobe epilepsy [49]. After seizures, the JNK signaling pathway is significantly activated in neurons [23]. Further studies have found that activation of the JNK signaling pathway has a certain regulatory effect on the expression and function of ENT1 in mouse myeloid leukemia cells and other models [25, 26]. To clarify the relationship between JNK and ENT1 in the epilepsy model, we established a pilocarpine‐induced epilepsy rat model and verified it by Western blot, immunohistochemical staining, and immunofluorescence staining. Previous tests have confirmed that ENT1 levels significantly increase during seizures and peak at 24 h [27, 50]. Therefore, 24 h after the successful establishment of the epilepsy model was also selected as the time point to observe the changes in various indices. Western blot analysis showed that the expression levels of ENT1 and p‐JNK were significantly lower in the SP600125 group than in the EP group, indicating that inhibition of the JNK signaling pathway can reduce the expression level of ENT1. The number of positive cells was counted and the average fluorescence intensity of ENT1 and p‐JNK was assessed in the CA1, CA3, and DG areas of the rat hippocampus through immunohistochemical and immunofluorescence staining. These results were found to be consistent with those obtained by Western blot analysis. Staining revealed that ENT1 was mainly expressed on the cell membrane, while p‐JNK was mainly expressed in the nucleus and part of the cytoplasm. Both were co‐expressed with MAP2, indicating that ENT1 and p‐JNK were expressed on rat hippocampal neurons.

This study used an SP600125 inhibitor in a rat model of acute epilepsy to modulate neurotransmitter release and change seizure intensity, thereby elucidating the central role of neurotransmitter balance in the occurrence and development of epilepsy. We observed that the seizure latency of the SP600125 group was significantly longer than that of the EP group, and the number of seizures within 1 h was significantly reduced. Hippocampal extracellular fluid was collected from six rats before and after seizures for detection by microdialysis combined with LC‐MS. Statistical analysis confirmed that adenosine and glutamate levels in the extracellular fluid of the hippocampus increased significantly after seizures in rats. The increase in adenosine levels may be related to its epileptic protective effect. By increasing the concentration of extracellular adenosine to terminate the ongoing seizures and change the pattern of subsequent seizures, epileptic activity was attenuated [51]. Early studies confirmed that in the epileptic cat model established by bicuculline, the extracellular adenosine concentration in brain tissue during epilepsy is 2–6 times higher than the normal value [52]. The same upward trend has also been observed in patients with epilepsy [53, 54]. Increased levels of the excitatory neurotransmitter glutamate are closely associated with the occurrence and persistence of seizures [55, 56]. Although adenosine can inhibit the release of glutamate by acting on the A1 receptor of the presynaptic membrane, the inhibitory effect of extracellular adenosine on glutamate cannot completely offset the increase in glutamate during acute seizures. In this experiment, only the levels of adenosine and glutamate in the extracellular fluid of the hippocampus were measured at 24 h. The lack of analysis over multiple time points to understand the dynamics of extracellular adenosine and glutamate is the shortcoming of this experiment.

The difference in the levels of adenosine and glutamate in the hippocampal extracellular fluid between the SP600125 and EP groups confirmed that the SP600125 inhibitor could significantly increase the concentration of adenosine in the hippocampal extracellular fluid of rat model, which also shows that in the pathological state of epilepsy, the direction of adenosine transport by ENT1 is mainly from extracellular to intracellular to reduce the content of adenosine in the extracellular fluid and promote the balance of intracellular and extracellular adenosine. The results showed that glutamate levels increased significantly in the EP group but decreased significantly in the SP600125 group, indicating that the increase in adenosine concentration in rat extracellular fluid could inhibit glutamate release and reduce the concentration of extracellular glutamate (Figure 1C).

## 5 Conclusion

After seizures, the JNK signaling pathway is significantly activated in neurons, and the JNK‐specific inhibitor SP600125 can specifically inhibit the JNK signaling pathway, reduce the expression of the ENT1 transporter, and reduce the seizure intensity in experimental rats. The mechanism is related to the transport of adenosine from the extracellular space to the intracellular space by ENT1 during epileptic states. Inhibition of ENT1 can increase the concentration of adenosine in the extracellular fluid of the hippocampus. The increase in adenosine concentration stopped glutamate from being released and reduced the amount of glutamate in the outside of the cell.

## Author Contributions

**Shun Liu:** conceptualization, methodology, investigation, formal analysis, writing – original draft. **Zhong Luo:** visualization, investigation, funding acquisition. **Fangjing Li:** resources, supervision. **Lijia Zhang:** validation. **Mingxiang Xie:** conceptualization, supervision, writing – review. **Juan Yang:** acquisition, writing – review. **Zucai Xu:** funding acquisition, resources, supervision, writing – review and editing.

## Ethics Statement

We confirm that we have read the journal's position on ethical publishing issues and confirm that this report complies with these guidelines.

## Conflicts of Interest

The authors declare no conflicts of interest.

## Data Availability

The datasets used and/or analyzed during the current study are available from the corresponding authors on reasonable request.
